# Physical Background of the Disordered Nature of “Mutual Synergetic Folding” Proteins

**DOI:** 10.3390/ijms19113340

**Published:** 2018-10-26

**Authors:** Csaba Magyar, Anikó Mentes, Erzsébet Fichó, Miklós Cserző, István Simon

**Affiliations:** 1 Institute of Enzymology, Research Centre for Natural Sciences, Hungarian Academy of Sciences, Magyar Tudósok krt. 2, H-1117 Budapest, Hungary; magyar.csaba@ttk.mta.hu (C.M.); mentes.aniko@ttk.mta.hu (A.M.); ficho.erzsebet@ttk.mta.hu (E.F.); cserzo.miklos@ttk.mta.hu (M.C.); 2 Department of Physiology, Faculty of Medicine, Semmelweis University, Tűzoltó u. 37-47, H-1094 Budapest, Hungary

**Keywords:** dehydron, homodimer, hydrogen bond, inter-subunit interaction, intrinsically disordered protein, ion pair, mutual synergistic folding, solvent-accessible surface area, stabilization center

## Abstract

Intrinsically disordered proteins (IDPs) lack a well-defined 3D structure. Their disordered nature enables them to interact with several other proteins and to fulfil their vital biological roles, in most cases after coupled folding and binding. In this paper, we analyze IDPs involved in a new mechanism, mutual synergistic folding (MSF). These proteins define a new subset of IDPs. Recently we collected information on these complexes and created the Mutual Folding Induced by Binding (MFIB) database. These protein complexes exhibit considerable structural variation, and almost half of them are homodimers, but there is a significant amount of heterodimers and various kinds of oligomers. In order to understand the basic background of the disordered character of the monomers found in MSF complexes, the simplest part of the MFIB database, the homodimers are analyzed here. We conclude that MFIB homodimeric proteins have a larger solvent-accessible main-chain surface area on the contact surface of the subunits, when compared to globular homodimeric proteins. The main driving force of the dimerization is the mutual shielding of the water-accessible backbones and the formation of extra intermolecular interactions.

## 1. Introduction

Since the millennium it has been clear that Anfinsen’s long-standing paradigm that was alleged to be valid for all proteins: “Protein structure is uniquely determined by its amino acid sequences” [1,2] is only valid for a specific subclass of proteins, while the rest of the proteins, termed intrinsically disordered proteins (IDPs), have no permanent 3D structures [3,4,5,6]. In our earlier effort to identify the physical background of protein disorder, the lack of sufficient pairwise interaction energy between the residues to ensure a stable 3D structure was pinpointed. When this energy is not enough to compensate the entropy-related free energy loss in the course of the formation of a unique structure, intrinsically disordered proteins are witnessed [7]. It has been shown that this pairwise energy can be calculated from the amino acid sequences without any structural information. On this basis we developed a widely used method, IUPred, to predict disordered proteins or protein segments from local composition data [8]. Another application of the estimation of the pairwise interaction energies led us to recognize the physical properties of the binding regions of disordered proteins, which can bind to ordered proteins [9]. When certain segments of a disordered protein interact with an ordered protein structure, part of their interactions will be manifested through elements of this stable globular protein having enough pairwise energy to stabilize their structures, i.e., to be folded, on the surface of ordered proteins. The contribution of a single residue depends only on the composition of the surrounding residues. Since ordered proteins have different amino acid compositions to disordered proteins, the resulting interaction energies of the residues at the contact surface can stabilize the structure (coupled folding and binding).

On the basis of this phenomenon, a binding site prediction method, termed ANCHOR, was developed [10]. These interacting segments generally appeared as short motifs of polypeptide chains (ELMs) [9,10]. More recently, the upgraded version of IUPred and ANCHOR were combined into a new server called IUPred2A [11].

**Figure 1:**
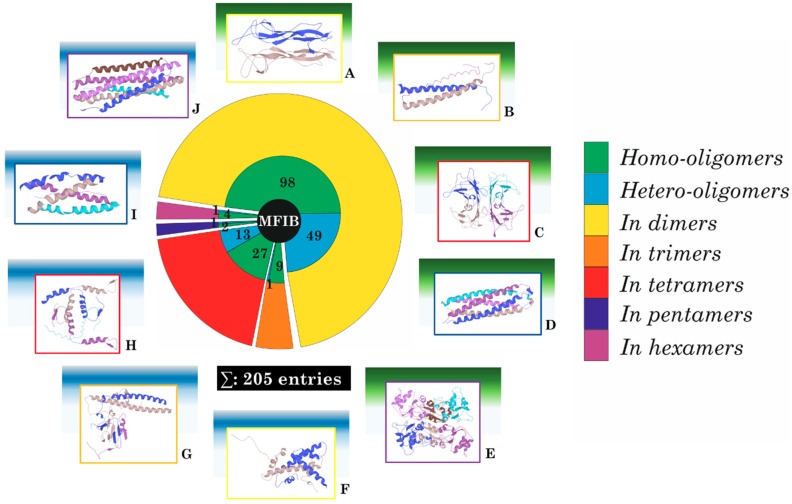
Oligomeric states in the MFIB database with example complexes. (**A**: 1BET, nerve growth factor (*Mus musculus*); **B**: 1AQ5, assembly domain of cartilage oligomeric matrix protein (*Gallus gallus*); **C**: 1GKE, Transthyretin (*Rattus norvegicus*); **D**: 1MZ9, assembly domain of cartilage oligomeric matrix protein (*Mus musculus*); **E**: 1NPK, nucleoside diphosphate kinase (*Dictyostelium discoideum*); **F**: 5GT0, H2A-H2B histone dimer, containing histone variants H2A type 1-A and H2B type 1-J (*Homo sapiens*); **G**: 2AZE, Rb C-terminal domain bound to an E2F1-DP1 heterodimer (*Homo sapiens*); **H**: 2NB1, p63/p73 hetero-tetramerization domain (*Homo sapiens*); **I**: 1VZJ, The synaptic acetylcholinesterase tetramer assembled around a polyproline-II helix (*Homo sapiens*); **J**: 1G2C, respiratory syncytial virus fusion protein core (*Homo sapiens*).

While this phenomenon appeared to be general, over the years the number of “exceptions” increased, suggesting that the insufficient pairwise energy calculated by the IUPred algorithms was only valid for certain intrinsically disordered proteins and protein segments (IDSs), and that another kind of IDP and IDS also existed. Even in the early age of IDP studies, there was sporadic information that some IDPs exhibit mutual folding and binding together with other IDPs, without the help of already stable proteins or other stable macromolecules [12,13]. For example, NCDB segments of CBP form a complex with the ACTR domain of p160, see: protein data bank (PDB) entry 1kbh [14] or region C of WASP is I complex with the GBD segment of WASP [15]. In these examples, the interacting parts of the disordered proteins were not ELM sized, but rather have structural domain sizes [16]. In many cases the interacting disordered protein segments were alike, forming homodimer or homo-oligomers. Here the coupled folding and binding should not appear due to the difference in residue composition, as in the case of ELMs stabilized on the surface of an ordered protein. Therefore there should be another mechanism for coupled folding and binding than the one we can recognize by ANCHOR. Since macromolecular interactions are part of almost all the activity of disordered proteins, a new mechanism for coupled folding and binding, where there is no stable template to use, define a new subset of IDPs. Despite the sporadic information about these interactions, not too many of this kind of complexes were reported in the literature [17,18]. Therefore we performed a detailed analysis of several databases and on the scientific literature and collected information on these complexes and created the Mutual Folding Induced by Binding (MFIB) database [19]. These complexes exhibit large structural variations (see Figure 1).

Almost half of the MSF-complexes are homodimers, but there is a significant amount of heterodimers and other oligomeric states, including homo- and heterotetramers, as well as trimers, pentamers, and hexamers. To explore the unique features of the entries in the MFIB database and pinpoint those characters that differ between these entries and those of those disordered segments that can participate in coupled folding and binding with already stable proteins, we created the Disordered Binding Site (DIBS) database of the latter complexes [20]. Currently, a publication of the comparison of the structural differences of proteins of the MFIB and DIBS databases is in progress [21].

The elements of the pairwise interaction matrices used in the IUPred and ANCHOR algorithms were derived from the structure data of folded globular proteins, therefore this data includes the free energy from the average hydration of the residues in these proteins. We showed that this is similar for most globular protein, therefore a fair free energy contribution of a particular residue can be calculated from the composition of the rather large polypeptide segment centered by the particular residue, using the pairwise energy interaction matrix [7]. In the IUPred algorithm, when a particular residue is processed, whether it belongs to an ordered segment or a disordered one, the interaction of this residue in question and all other residues in a large surrounding region are considered. Therefore this calculated energy value has to be the same for all permutations of the residues of the segments located at both sides of the center residue, until or unless the compositions of the segments are changed. The amino acid sequences of proteins that have stable folded structures evolved in such a way that the side chains together shield the backbone from water, which minimizes the energetically unfavored water-accessible area on the polypeptide backbone. In this work we show that this statement is not valid for the disordered proteins listed in MFIB.

We investigated whether the interacting regions of these proteins can be identified based on their location in the whole polypeptide chain, on their biased amino acid composition or on specific physical properties. We discovered that their most unique characteristic is the high water accessibility of their peptide backbone, compared to the water accessibility of the folded proteins, which have similar amino acid compositions.

## 2. Results

### 2.1. Sequence-Based Analysis

**Figure 2:**
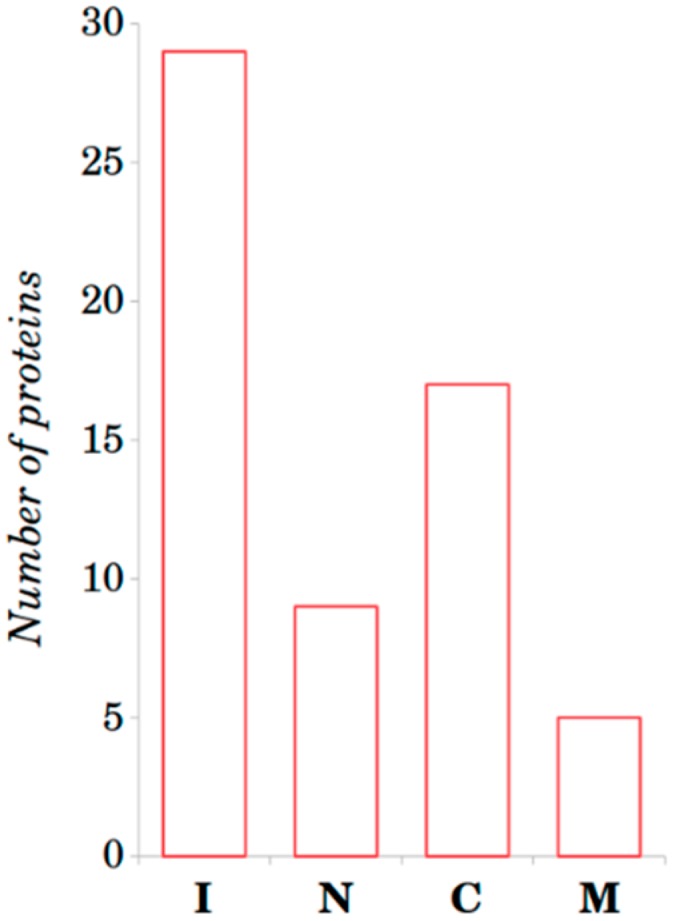
Distribution of MFHD PDB segments in the full UniProt sequences. (I: Amino acid sequence from UniProt is identical with amino acid sequences of MFHD PDB segment amino acid sequences; N: MFHD PDB segment is located in N-terminus of the amino acid sequences from UniProt; C: MFHD PDB segment is located in C-terminus of the amino acid sequences from UniProt; M: MFHD PDB segment is located in middle of the full amino acid sequence from UniProt).

In this study, homodimeric protein complexes from MFIB were analyzed regarding sequence and structural properties. First we checked the location of the MFIB homodimeric dataset (MFHD) PDB segments with a known 3D structure in the full UniProt protein sequences. In some cases, the MFHD PDB segments were located near the N-terminus, near the C-terminus, in the middle of the sequence or they were identical with the full sequence (Figure 2).

**Figure 3:**
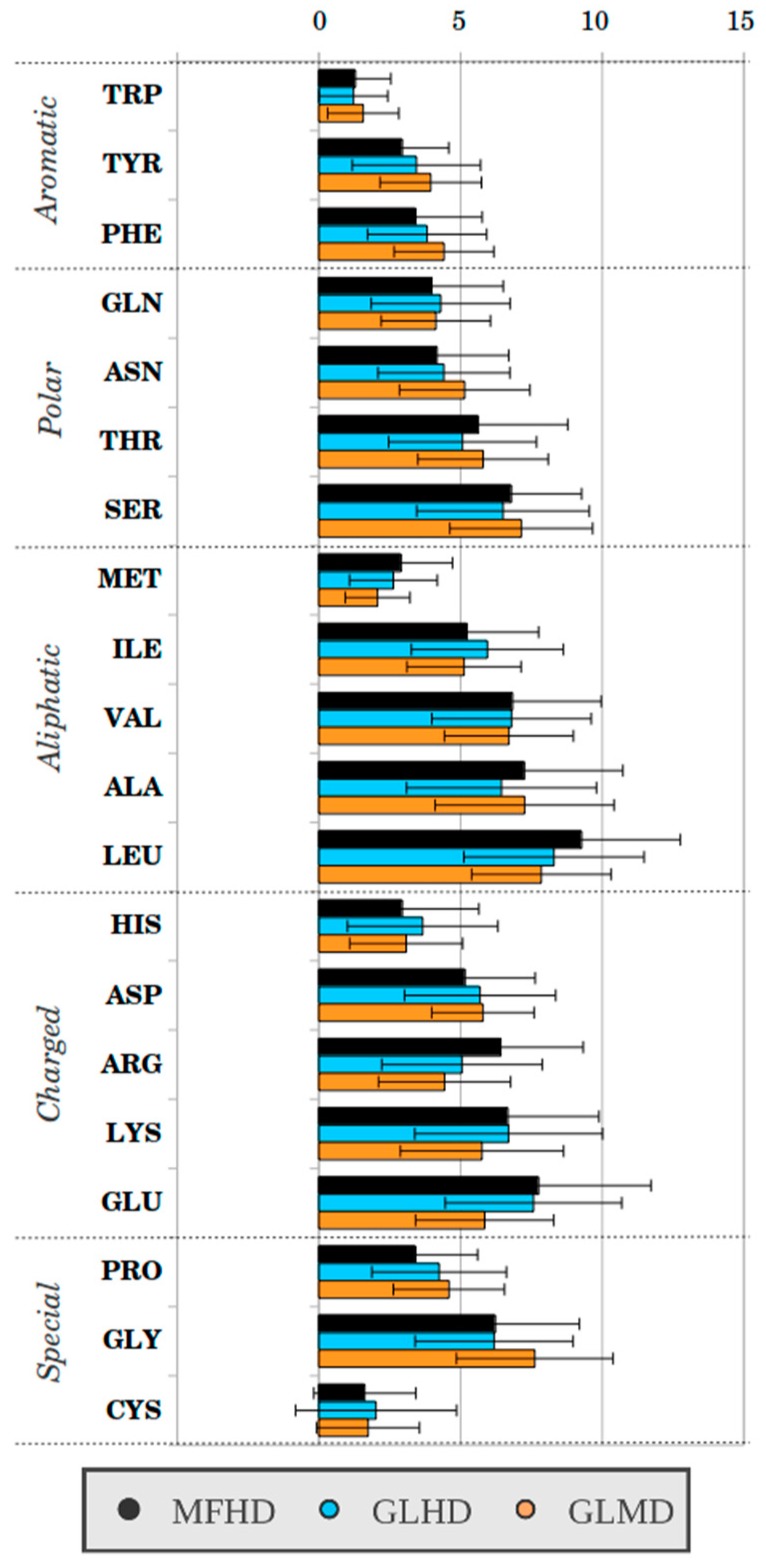
Sequence properties of MFHD, GLHD, and GLMD proteins (For values, see Table S2).

**Figure 4:**
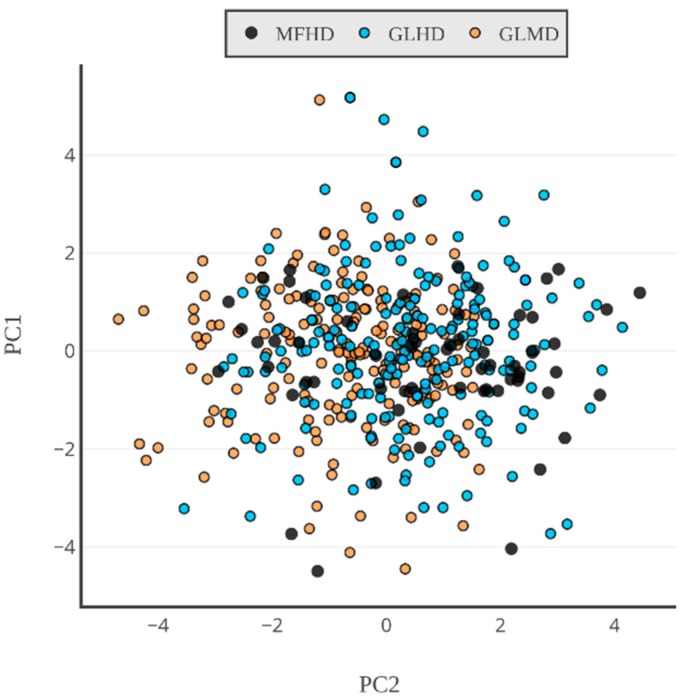
PCA ordination of the proteins from MFHD, GLHD, and GLMD based on their amino acid compositions (for values, see Table S3).

We examined the residue composition of the MFHD proteins (Figure 3, Table S2), which were compared with two reference datasets, the globular homodimeric dataset (GLHD) and the globular monomeric dataset (GLMD, see Section 4). To better understand the amino acid composition of the sequences, it was depicted by principal component analysis (PCA) (Figure 4, Table S3). PCA showed that the amino acid composition of the MFHD proteins did not differ significantly from the amino acid composition of the globular proteins (GLHD, GLMD). The PCA also demonstrated that MFHD formed a diverse group based on their amino acid composition.

We investigated the MFHD with several protein disorder predictors (IUPred, ESpritz, GlobPlot, VSL2b, MobiDB Lite, MetaDisorder) [8,22,23,24,25,26], which worked well on the IDPs listed in DIBS, but did not recognize the polypeptide of MFHD complexes and other members of the MFIB database as disordered proteins. All methods predicted less than 30% of the protein residues as disordered, while the IUPred long/short methods, relying on a physical basis, predicted only 8 and 10% of the protein residues as disordered, respectively (for values, see Table S4). Other prediction methods based on amino acid composition bias also failed to detect MFHD PDB segments. Methods developed from the DAS and DAS-TMfilter [27,28] algorithms were tested on the dataset.

### 2.2. Structure-Based Analysis

We will use the term “interface” for the contact surface area of the two identical subunits in the dimeric structures. In cases where the term “monomeric structure” is used, calculations were carried out on structures from which the second chain was deleted since the PDB files contained dimer forms of the complexes. Residues belonging to the interface region were identified based on solvent accessible surface area (SASA) calculations. All-atom SASA values were calculated for the residues. Residues where the SASA value calculated from the dimer form were less than or equal to 20% of its counterpart from the monomeric structure defining the interface. We found that on average there were 26.4 interface residues per polypeptide-chain in the MFIB homodimeric dataset and 21.0 interface residues per polypeptide-chain in the reference globular homodimeric dataset. Considering the average size of the protein, this means that 27.13% of all residues in the MFHD and 22.34% of all residues in the GLHD belonged to the interface region. The higher value obtained for the MFIB homodimeric structures indicates that inter-subunit interactions may play an essential role in the stabilization of MFHD proteins.

**Figure 5:**
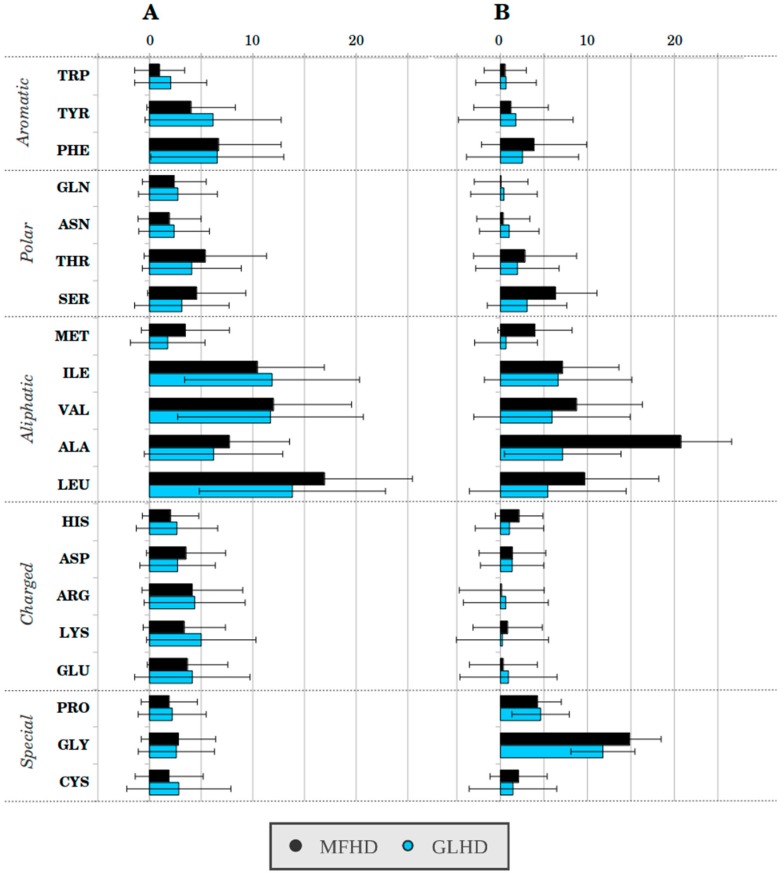
Amino acid composition of interface region (**A**) and RSAMPs (**B**) of the MFIB and globular homodimeric datasets (For values, see Tables S5 and S6).

We were looking for residues in the interface that have solvent-accessible spots in their main-chain in the monomeric structure, which become buried in the dimeric structures. We identified residues where the main-chain SASA in the dimeric form was less than 20% of the monomeric form value. Only residues with exposed main-chains, with a relative main-chain SASA larger than 0.2 in the monomeric structure, were taken into account. These residues with solvent-accessible main-chain patches (RSAMPs) were believed to be the main driving force of the dimerization of the disordered polypeptide chains collected in the MFIB database. We found a total of 183 such residues in the MFHD proteins; all structures contained at least one such residue. This was 3.14% of all residues. Considering that 27.13% of the residues were forming the interface, this means that 11.57% of the MFHD interface residues were RSAMPs. In the GLHD, 40.83% of the proteins did not contain such residues, on average 1.56% of all residues were RSAMPs. Since 22.34% of the residues form the interface, only 6.98% of the interface residues were RSAMPs. We calculated the average solvent-accessible surface area of the main chains. In the MFHD, the average solvent-accessible, main-chain area belonging to the interface region was 1154.56 Å^2^ per polypeptide-chain, while in the GLHD this value was 790.54 Å^2^. We can see that in the case of MFIB proteins a larger main-chain surface area is solvent accessible, which is energetically not favorable. The amino acid composition of the interface region and RSAMPs of the MFHD and GLHD complexes can be seen in Figure 5, Tables S5 and S6. Alanine and glycine were the most abundant residues under RSAMPs, which might be responsible for the higher solvent accessibility of the main chain in the MFHD. In the interface region, aliphatic residues are predominant. In the MFHD this was 50.6%, while in the GLHD 45.4% of the interface residues were aliphatic, making inter-subunit hydrophobic interactions even more prominent in MFIB proteins.

We determined the secondary structural propensities in the MFHD, GLHD, and GLMD. We found that in the MFHD a significantly higher percentage of residues (39.4%) belonged to α-helices when compared to GLHD and GLMD (39.4% and 27.9%). In the MFHD, 21.2% of the residues belong to β-sheets, while in the GLHD and GLMD this value was 27.1% and 28.3%, respectively. The MFIB proteins show higher helical propensities than globular proteins.

We identified the hydrogen bonds formed between the two subunits. In the MFHD 6.97 inter-subunit H-bonds per structure were found, while in the GLHD this was only 4.58. Furthermore we identified underwrapped hydrogen bonds that are not well-enough shielded from the solvent, called dehydrons, in all structures [29]. In the MFHD we found 3.11 dehydrons per polypeptide chain under the inter-subunit H-bonds, while only 2.18 were found in the GLHD. Contrary to these results is the average wrapping of inter-subunit H-bonds, which was 16.0 for the MFHD and 13.6 for the GLHD. Although there were more dehydrons—i.e., underwrapped H-bonds—in the MFHD, the average wrapping value was still higher.

Due to the large difference found in the inter-subunit H-bonds, other inter-subunit interactions were also investigated. First we identified inter-subunit ion-pairs. We found that in the MFHD there were 1.17 inter-subunit ion-pairs on average, with only 0.66 in the GLHD. Charged residues tend to occur at the surface due to the desolvation of buried charges being energetically not favorable. Charged residues buried either in the interior of a protein or in the interface region of the dimeric structure should form ion pairs in order to compensate the desolvation penalty through favorable electrostatic interactions. Since the occurrence of charged residues is a bit higher in the interface region of the GLHD (16.2% vs. 14.7%), the lower number of inter-subunit ion-pairs was unexpected. We already noted in an earlier publication that inter-subunit ion pairs might contribute to the stabilization of proteins [30].

Stabilization centers (SCs) are pairs of residues involved in more than average long-range interactions [31]. These residue clusters are believed to contribute to the stabilization of protein structures through the cooperativity of the individual interactions [32,33]. The stabilization centers formed between different polypeptide chains can contribute to the stabilization of a protein complex [34]. We identified inter-subunit SCs in both the MFHD and GLHD. The two residues that form a stabilization center are called stabilization center elements (SCEs). We identified the SCEs belonging to the interface. In the MFHD, 3.86% of all residues form inter-subunit SCs, that is on average 14.22% of the interface residues form inter-subunit SCs. In the GLHD, only 1.83% of the residues belong to inter-subunit SCs. This means that only 8.19% of the interface residues form inter-subunit SCs. In MFIB dimers, the inter-subunit SCs were much more frequent than in the GLHD. We investigated whether SCEs overlap with RSAMPs or whether they are segregated. We found that there was a significant overlap, as 29.51% of the RSAMPs were SCEs in the MFHD. In the GLHD, we obtained a similar value of 29.19% for the overlap. 

## 3. Discussion

In a recent study, we compared the residue composition of IDPs from the MFIB with complexes from the DIBS and other human protein databases [21] and we found that the composition of MFIB complexes was significantly different from that of the DIBS and only slightly different from that of human proteins. IDPs from the DIBS database are capable of coupled binding and folding on the surface of ordered proteins and can be predicted through bioinformatics methods like the ANCHOR algorithm, which is based on the different residue composition of the disordered monomer and the disordered–ordered protein complex. Therefore, in this work we studied MSF-homodimers to exclude this explanation for the case of mutual synergistic folding. We observed that in some cases the interacting segment of MFIB homodimers was the full polypeptide chain, while in other cases only a part of the chain was involved in the dimerization (Figure 2). We showed that they could be an order of magnitude longer than ELMs, which can be recognized by ANCHOR in other proteins.

In our current study the residue composition of the homodimeric complexes from the MFIB was determined and compared with that of homodimeric and monomeric globular proteins in similar amino acid sequence lengths (Figure 3 and Figure 4). Our results showed that the IDPs listed in the MFIB had a similar amino acid composition to that of globular proteins. The PCA showed that the globular (GLHD, GLMD) and the MFHD proteins were not distinguishable. Although the points belonging to the complexes in the PCA figure were not certainly clustered, suggesting that MFHD is a distinct subgroup of IDPs. This was confirmed by the comparison of MFHD with the UniRef50 database, which showed that the main part of MFHD belongs to a distinct cluster and there is no significant similarity between their Pfam domains.

We investigated the MFHD with several protein disorder predictors (IUPred, ESpritz, GlobPlot, VSL2b, MobiDB Lite, MetaDisorder), which work well on the IDPs listed in DIBS. These methods did not recognize the full-length polypeptide chains of the MFHD complexes and other members of the MFIB database as disordered proteins. Since the disorder predictors IUPred and ANCHOR rely exclusively on solid physical principles, these methods were used to discover the physical principles behind the disordered character of the protein and the origin of the coupled folding and binding of the homodimers in the MFIB database. Our current study indicated that in the case of MFHD, the IUPred algorithm using its standard 20 × 20 pairwise free energy matrix overestimated the stabilizing energy because the energetically-unfavorable large solvent-accessible surface area of the peptide backbone in single protein chains resulted in less stabilizing energy. This can explain why these proteins were disordered in monomeric form. On the one hand, members of the MFIB dataset can be disordered for similar reason than other disordered proteins. That is, the sum of their pairwise interaction enthalpy did not compensate the free energy contribution of the entropy loss during folding. However, this is not the consequence of the amino acid composition of these polypeptides. Pairwise interactions of residue pairs, which have backbone parts not sufficiently shielded from the solvent, contribute less enthalpy to the stabilization than that found in globular proteins, from which the standard 20 × 20 pairwise free energy matrix was derived. Therefore, by using the free energy matrix in IUPred, we overestimated the stabilizing free energy of the proteins listed in the MFIB. This is why the IUPred algorithm predicted these monomers as structured proteins, while the experiments showed that they are disordered in their monomeric form [16].

We can conclude that the residue composition of MFHD is rather similar to that of the globular proteins (GLHD and GLMD), we were looking for structural differences among them. We found that the interface region had more residues in the MFHD than in the GLHD. MFIB homodimeric proteins had a larger solvent-accessible main-chain surface area in the interface when compared to globular homodimeric proteins. The polypeptide backbone of MFHD proteins was more accessible for water than in globular proteins. During dimerization, the solvent-accessible surface area of the backbone decreased and a high number of inter-subunit interactions (H-bonds, ion-pairs and stabilization centers) formed, leading to the stabilization of the of the disordered polypeptide-chains, enabling an ordered structure of MFIB proteins in the dimeric form. The driving force of the dimerization was the mutual shielding of the water-accessible backbones and the formation of extra intermolecular interactions.

## 4. Materials and Methods

Filters were applied to the homodimeric structures of the MFIB database. A reference dataset was created from homodimeric globular proteins, where the monomeric form was also globular in itself. Another reference dataset was created from monomeric globular proteins.

All homodimeric structures were collected from the MFIB database, and the modified PDB files were used. Entries belonging to the “coils and zippers” structure class were discarded since structures belonging to this class are both sequentially and structurally different from other homodimers. It is evident that a structure like a leucine-zipper cannot exist in monomeric form, thus no reference dataset can be created from “coils and zippers” where the monomer is not disordered in itself. A contact map matrix for all remaining structures was created. Entries with unusual contact maps were manually inspected. After inspection, the following entries were discarded: 2adl, 1r05, 4ath, 1aa0, 4w4k, 1ejp, resulting in a dataset of 60 homodimeric structures (Table S1). Heteroatoms were deleted from the structure. This dataset was referred to as MFIB homodimeric dataset (MFHD). We checked the secondary structure of the databases using the DSSP 2.0.4 program [35]. We found that 39.4% of the residues belonged to α-helices and 21.2% to β-sheets. The size distribution of the dataset was investigated. We counted the number of residues belonging to the N, N + 20 intervals. We found that the 140–240 interval was predominant, thus the reference datasets were created according to this size distribution. 

A non-redundant reference dataset was created from homodimeric globular proteins. All homodimeric structures within the 140–240 amino acid size range were collected from the non-homologous PDB_Select database as of November, 2017 [36]. Structures containing coiled-coil structural elements identified with the Socket 3.0.3 program were excluded from the dataset [37]. The proper quaternary structure of the homodimers was created according to the BIOMT records of the PDB files. Entries with the following PDB ligand summary “ids” of large molecular sizes ligands and cofactors were discarded from the dataset because they could significantly alter the results of the solvent-accessible surface area calculations (017, 1BG, 1PE, 1PG, 5GP, C2E, FAD, HEC, KI1, MYA, MYR, MYS, NER, O8N, OLC, P33, P6G, PE5, UNL). Heteroatoms were deleted from the remaining structures. This procedure resulted in a list of 218 protein structures. This dataset was referred to as the globular homodimeric dataset (GLHD). For the PDB codes, see Table S1. According to DSSP, 27.9% of the residues belonged to α-helices and 27.1% to β-sheets.

An additional non-redundant reference dataset of the monomeric structures in the 140–240 amino acid size range containing only one structural domain was created from the PDB_SELECT database. The initial database was filtered by size and monomeric state criteria. All entries proved to be single domain according to the DDomain program using authors-trained parameters [38]. This dataset was referred to as the globular monomeric dataset (GLMD) and contained 191 entries (Table S1). According to DSSP, 24.9% of the residues belonged to α-helices and 28.3% to β-sheets.

Differences in the amino acid composition of the proteins sequences from the MFHD, GLHD, and GLMD datasets were revealed by principal component analysis (PCA) ordination using the plotly software according to Raska [39]. 

Hydrogen bonds were identified using the find_pairs command of PyMOL using 3.5 Å distance and 45 degree angle criteria between the donor and acceptor groups [40]. The calculation of the wrapping of hydrogen bonds and the identification of dehydrons was performed with the dehydron_ter.py program [41].

Stabilization centers (SCs) are pairs of residues, called stabilization center elements (SCEs), which are involved in several long-range interactions. These residues can be identified with our publicly available web server at http://scide.enzim.hu [42]. 

The solvent-accessible surface area (SASA) was calculated using the FreeSASA 2.03 program [43]. A residue was classified as buried when its relative SASA was below or equal to 0.2. Residues with a relative SASA value of over 0.2 were considered as exposed. A residue was classified as part of the interface region when its all-atom SASA calculated from the dimeric structure was less than 20% of the value calculated from the monomeric structure (created by deleting the second chain from the PDB file). 

Ion-pairs were defined as pairs of negatively and positively charged residues, where the distance between the charged groups was equal to or less than 4 Å [44]. Ion pairs were identified using our own C++ program.

## Supplementary Materials

Supplementary materials can be found at http://www.mdpi.com/1422-0067/19/11/3340/s1. Table S1. List of PDB entries in the MFHD, GLHD, GLMD datasets. Table S2. Average amino acid sequence composition of proteins from MFHD, GLMD, and GLHD. Table S3. Amino acid sequence composition of proteins from MFHD, GLMD, and GLHD for PCA. Table S4. Disorder content by various predictors. Table S5. Average amino acid composition of interface region of the proteins from MFHD and GLMD. Table S6. Average amino acid composition of RSAMPs of the proteins from MFHD and GLMD.

## Abbreviations

IDPs	Intrinsically disordered proteins
MFIB	Mutual Folding Induced by Binding database
DIBS	Disordered Binding Site database
ELMs	Short motifs of polypeptide chains
MFHD	MFIB homodimeric dataset
MSF	Mutual synergistic folding
GLHD	Globular homodimeric dataset
GLMD	Globular monomeric dataset
PCA	Principal component analysis
PDB	Protein data bank
RSAMPs	Residues with solvent accessible main-chain patches
SC/SCE	Stabilization centers/stabilization center elements
SASA	Solvent accessible surface area
